# Outbreak of Pertussis in a School and Religious Community Averse to Health Care and Vaccinations — Columbia County, Florida, 2013

**Published:** 2014-08-01

**Authors:** James Matthias, Cristina Dusek, P. Scott Pritchard, Laura Rutledge, Paula Kinchen, Mark Lander

**Affiliations:** 1 Bureau of Epidemiology, Florida Department of Health; 2 Bureau of Communicable Diseases, Florida Department of Health; 3 Florida Department of Health in Columbia County

On August 30, 2013, the Florida Department of Health in Columbia County was notified of a *Bordetella pertussis* laboratory-positive unimmunized child attending a local charter school (316 students from pre-K through 8th grade) in a large religious community averse to health care and vaccinations. Kindergarten immunization records showed that only five (15%) of 34 students were fully immunized with pertussis-antigen–containing vaccines. In seventh grade, only one (5%) of 22 students was fully immunized with pertussis-antigen–containing vaccines. Of the children who were not fully immunized in these two grades, 84% had religious exemptions (1).

Interviews confirmed that a sibling of the patient had symptoms consistent with pertussis. By September 3, two additional children from the same school were confirmed by polymerase chain reaction to have pertussis. On September 12, the Florida Department of Health in Columbia County declared a communicable disease emergency; children with cough illness were excluded from school, and reentry required an evaluation by a health care provider. After this declaration, 38 additional students were excluded. Prophylaxis or treatment with antibiotics following current guidelines were provided to patients and household contacts (2). The local health department offered to provide these services free of charge to persons without health care coverage. Pertussis vaccine administered at the health department was available; however, fewer than five persons from the community used this opportunity for vaccination.

An investigation was conducted to determine disease incidence within the community and measures to control the spread of the disease. Medical record review and household interviews were conducted for children excluded or absent from the charter school because of cough illness. Cases were classified as confirmed or probable using the Council of State and Territorial Epidemiologists case definitions (3). Additionally, a suspected case was defined as either 1) a cough illness that lasted 7–13 days but did not meet either the confirmed or probable case definition, or 2) an illness in a person who received treatment for pertussis without additional clinical details. Case onset dates, vaccination status, demographics, and school attack rates were evaluated.

A total of 109 cases were identified in the community, of which eight were classified as confirmed (five by laboratory confirmation), 61 were probable, and 40 were suspected. Of 316 students and 16 teachers, 94 (30%) students and one teacher had illnesses that met one of the three case definitions. Fourteen cases, including three in infants, were in household contacts of ill charter school students. None were hospitalized. Only one confirmed or probable case was reported in a person who reported having received any vaccination against pertussis. Nearly 90% of persons with illnesses meeting one of the case definitions were evaluated and prescribed antibiotics consistent with current guidelines by a single pediatrician in the course of his normal practice; laboratory testing was performed in only nine of 109 cases (2). Attack rates were highest among the youngest students (57.1% among pre-kindergarten students) and decreased with increasing age (to 14.3% among 8th-grade students).

In vaccine-averse communities, controlling vaccine-preventable disease outbreaks is challenging, particularly when susceptible community members have prolonged contact in multiple settings. Local public health agencies need to identify and collaborate with institutions and health care resources to reduce morbidity from vaccine-preventable diseases in communities where a substantial number of persons do not have immunity. Physicians should pursue laboratory testing for pertussis in patients with symptoms consistent with the disease. However, physicians also need to understand the importance of reporting presumptive pertussis cases, even without laboratory confirmation, to public health departments.
